# Reasons for Inconsistent Condom Use Found as Answers to a Multiple Response Question: A National Survey of Iranian Adults, 2013

**Published:** 2017-08-10

**Authors:** Moghaddameh Mirzaee, Yunes Jahani, Hamid Sharifi

**Affiliations:** ^1^ HIV/STI Surveillance Research Center, and WHO Collaborating Center for HIV Surveillance, Institute for Futures Studies in Health, Kerman University of Medical Sciences, Kerman, Iran; ^2^ Modeling in Health Research Center, Institute for Futures Studies in Health, Kerman University of Medical Sciences, Kerman, Iran; ^3^ Department of Biostatistics and Epidemiology, School of Public Health, Kerman University of Medical Sciences, Kerman, Iran

**Keywords:** Condom, Adults, Iran, Multilevel analyses, Multivariate

## Abstract

**Background:** The use of condoms is important for preventing Sexually Transmitted Infections (STIs). However, the prevalence of condom use is not satisfactory. The aim of this study was to assess the reasons for inconsistent condom use in Iranian adults.

**Study design:** Cross-sectional study.

**Methods:** Data were gathered through multi-stage sampling. Participants were enrolled from 13 provinces in Iran aged between 19 and 29 years. They had ever-extramarital sex and did not use condoms regularly in their sexual contacts, asked the reasons for inconsistent condom use.

**Results:** We had 3,246 adults, from which 635 (19.5%) had ever-extramarital sex. Among them, 495 (77.96%) did not use condoms with regularity. The reason frequently chosen for inconsistent condom use was ‘that it is not accessible’ (49.3%). The differences between the categories of some variables, according to the choice of reasons for inconsistent condom use, were significant (*P*<0.05): age, gender, knowledge of HIV, attitude towards HIV, knowing infected HIV person and alcohol or stimulant(s) used before sexual contact. According to multivariate multilevel logistic, the effect of gender was significant on most of reasons for inconsistent condom use (*P*<0.05).

**Conclusions:** Most of the reasons that were selected for inconsistent condom use were inaccessibility and not knowing that its use is essential. Hence, it is important to improve the knowledge of adults regarding STI/HIV and extend the locations of condom distribution. We should try to promote the culture of condom use as a routine.

## Introduction

Adult youths get involved in sexual behaviours that put them at risk for Sexually Transmitted Infections (STIs), such as HIV infection ^1, 2^. In Iran, extramarital sex is prohibited under Islamic religious and Iranian country law. However, apart from this issue, the prevalence of extramarital sex was estimated in special groups, such as internal long-distance truck drivers, married undergraduate university students, and males who are at risk of HIV between 0.01% up to 41.3% ^3-5^, but there is no study on general Iranian adult youths estimating its prevalence.

In countries other than Iran, the prevalence of extramarital sex (having more than one partner over the past 12 months) among married men and women 18–75 yr old is estimated at 2.2% in the national sample and 2.5% in urban samples in the United States ^6^. As estimated, 26%-50% of married men and 21%-38% of married women had extramarital sex ^7-9^. In sexually active adult youths, the most effective tool for preventing STI/HIV is the use of condoms during sexual contacts ^10-12^. But the consistency of condom use is not satisfactory in Iran because the prevalence of condom use in special groups like college students, female sex workers, and in sexually active male Injecting Drug Users (IDUs) was estimated at between 38.1%-83.3% ^13-15^. In sub-Saharan Africa and Asia, the prevalence of consistent condom use—according to partnership types—was estimated at between 8%-56% ^16^. Among female sex workers, university students and urban adolescents, such prevalence is estimated at 35%-68.7% ^17-19^. Some studies have carried out work on the estimated prevalence of condom use in people who engage in extramarital sex or on the association between the use of condoms and extramarital sex ^20, 21^. Again, many studies have investigated the factors that affect condom use or have investigated the factors that have relationships with it ^22-24^. Most of the studies focused on discriminant people in two groups, who are consistent and not consistent with condom use, and then surveyed the factors that can affect the odds of being consistent with condom use. There is an important reason why these people do not use condoms with regularity in their sexual contacts.

There are limited studies that have investigated the reasons for not using condoms with regularity in high-risk sexual behavioural people ^25, 26^. It is an important problem: Identifying these reasons, and by intervening in them, and improving the consistency of condom use. In this study, we investigated the reasons for inconsistent condom use as a multiple response question in Iranian adult youths who have had extramarital sex.

## Methods

### Study population and samples

This cross-sectional study was part of a big study, the questionnaire of which had many questions about knowledge, attitude and practice toward HIV in adults between 15–29 years. Practice questions were filled only by adults between 19–29 yr because of ethical considerations. That questionnaire had questions such as ‘Did you ever have extramarital sex?’ and ‘Did you use a condom regularity?’, and the reasons for inconsistent condom use were asked. Therefore, people who reported having had extramarital sex and not using condom regularity were included in our study.

The study population comprised all adult youths between 19 and 29 yr, living in Iran, who had ever had extramarital sex—which means in lifetime having sexual contact outside formal marriage—and were not using condoms with regularity.

Samples were enrolled into our study through multistage cluster sampling (Stage 1: Selection of the province; Stage 2: Selection of two cities from each province; Stage 3: Selection of some villages and some streets from selected cities; and Stage 4: Selection of adults from Stage 3). In total, 31 provinces in Iran were divided into three categories in accordance with the degree of literacy (low, moderate, high); thus, we sampled four, three and six provinces from categories with low, moderate and high literacy respectively. In total, 13 provinces were considered in our study.

In each province, we selected the capital city of the province, and one suburban city, along with some villages. These were divided into three categories in accordance with the development index. We chose one village from each category. The sample size in each province was 330; such that 220 samples of questionnaires were filled in the capital city and the remaining 110 were allocated to the suburb city, (77 questionnaires were filled in the suburb city and 33 allocated to villages). In all samples, half the questionnaires were filled by males and the other half by females. For sampling from the capital city, we considered the city as being formed of five regions. In each region, we selected two of the main streets and the suburban city was considered to comprise three regions. In each region, we selected two main streets. In villages, questionnaires were filled up in public places.

### Instrument

The instrument of measurement was a questionnaire that consisted of demographic items, knowledge, attitude, and practice towards HIV. Reliability and validity were measured earlier ^27^.

### Response variable

The response variable was the reasons for inconsistent condom use. Samples could select more than one choice; hence, it is considered as a multiple response variable. This variable had these choices:

1. It was not accessible
2. My partner objected
3. It is too expensive
4. I do not like it
5. I used something else
6. I did not think it was necessary
7. Other

### Statistical analysis

We described the distribution of samples in accordance with the variables by count and percentage. The reasons for inconsistent condom use were presented as a multiple response. Then the homogeneity of choosing the reasons for inconsistent condom use in sub groups of variables was conducted by marginal independent test. We used a multivariate multilevel logistic regression for calculating the adjusted odds ratio .We used the backward method and reported variables that were significant at 0.05 level. All analysis conducted in R software (Version 3.3.1).

### Ethical considerations

The method of gathering data was street-based. Interviewers verbally explained the goal of the survey and emphasized that their forms were with no names. Hence, participation was free for inclusion in the study. All participants signed their forms in accordance with their personal satisfaction. The Ethics Committee of the Kerman University of Medical Sciences vouched for the protocol of this study (Reference Number: K/93/205).

## Results

We had 3,246 adults aged between 19 and 29 yr, from which 635 (19.5%) had ever had extramarital sex. Among them 140 (22.04%) used condoms in contact sex with regularity. Hence, 495 (77.96%) of people did not use condom regularly and so were asked the reasons for inconsistent condom use. The rate of non-response was 13.2%.

Most of the adults were between 19 and 24 yr (52.9%), male (74.1%), single (66.3%), employed (55.4%) and university students or graduates (48.9%) (Table 1).

**Table 1:** Characteristics of the study population (n=495)

**Variables**	**Number**	**Percent**
Age (yr)		
19-24	262	52.9
25-29	233	47.1
Gender		
Female	128	25.9
Male	367	74.1
Marital Status		
Single	328	66.3
Married	143	28.9
Widowed/Divorced	24	4.8
Education		
Less to primary	79	16.0
Primary to high diploma	174	35.2
University student or graduate	242	48.9
Job		
Employed	274	55.4
Unemployed	221	44.6
Residence		
Urban	109	22.0
Rural	386	78.0
Knowledge toward HIV		
Low	227	45.9
Moderate	194	39.2
High	74	14.9
Attitude Toward HIV		
Negative	30	6.1
Neutral	207	41.8
Positive	258	52.1
HIV Testing		
Yes	115	23.2
No	380	76.8
Knowing infected HIV person		
Yes	51	10.3
No	444	89.7
Ever Used Methamphetamine		
Yes	107	21.6
No	388	78.4
Ever used alcohol or stimulant before sexual contact	
Yes	194	39.2
No	271	54.7
Don't remember	30	6.1
Ever used ecstasy		
Yes	56	11.3
No	421	85.1
Don’t remember	18	13.6
Age at first extramarital sexual contact		
≤20	310	62.6
>20	185	37.4
Ever used injected Stimulant drugs		
Yes	37	7.5
No	458	92.5

Knowledge and attitude towards HIV were mostly low (45.9%) and positive (52.1%), respectively. Most of the people having tested for HIV (23.2%) had also used methamphetamine (21.6%), ecstasy (11.3%), injected stimulant drugs (7.5%) and alcohol or some other stimulant before sexual contact (39.2%).

Table 2 shows the important reasons for inconsistent condom use was ‘that it is not accessible’ (49.3%) and after that ‘they think it was not necessary’ (41.4%).

**Table 2:** Reasons for inconsistent condom use in the study population (n=495)

**Reasons for inconsistent condom use** ^a^	**Number**	**Percent**
It was not accessible	244	49.3
It is too expensive	45	9.1
My partner objected	96	19.4
I do not like it	183	37.0
I used something else	28	5.7
I did not think it was necessary	205	41.4
Other	25	5.1
**Total**	826	166.9

^a^ This variable had multiple responses

The relationship between the variables and the reasons for inconsistent condom use has been reported in Table 3. The frequently selected reason for inconsistent condom use in adults aged between 19 and 24 yr and 25 and 29 yr was ‘inaccessibility of condoms’ (49.6%) and (48.9%) respectively. The other choices were different between the two groups of ages (*P*-value=0.002).

**Table 3:** Distribution of variables according to reasons for inconsistent condom use in the study population (n=495)

**Variables**	**Reason 1**		**Reason 2**		**Reason 3**		**Reason 4**		**Reason 5**		**Reason 6**		**Reason 7**		***P*** ** value**
	**n**	**%**	**n**	**%**	**n**	**%**	**n**	**%**	**n**	**%**	**n**	**%**	**n**	**%**	
Age (yr)															0.002
19-24	130	49.6	32	12.2	67	25.6	91	34.7	14	5.3	104	39.7	15	5.7	
25-29	114	48.9	13	5.6	29	12.4	92	39.5	14	6.0	101	43.3	10	4.3	
Gender															0.001
Female	39	30.5	9	7.0	40	31.2	33	25.8	7	5.5	63	49.2	7	5.5	
Male	205	55.9	36	9.8	56	15.3	150	40.9	21	5.7	142	38.7	18	4.9	
Marital Status															0.304
Single	166	50.6	33	10.1	57	17.4	123	37.5	20	6.1	126	38.4	20	6.1	
Married	70	49.0	9	6.3	33	23.1	52	36.4	8	5.6	66	46.2	3	2.1	
widowed/Divorced	8	33.3	3	12.5	6	25.0	8	33.3	0	0.0	13	54.2	2	8.3	
Education															0.187
Primary or less	43	54.4	7	8.9	12	15.2	31	39.2	0	0.0	34	43	3	3.8	
Primary to high diploma	98	56.3	14	8.0	34	19.5	63	36.2	12	6.9	67	38.5	6	3.4	
University student or graduate	103	2.6	24	9.9	50	20.7	39	36.8	16	6.6	104	43	16	6.6	
Job															0.448
Employed	137	50.0	24	8.8	43	15.7	100	36.5	14	5.1	118	43.1	13	4.7	
Unemployed	107	48.4	21	9.5	53	24.0	83	37.6	14	6.3	87	39.4	12	5.4	0.116
Residence															
Urban	191	48.6	30	7.8	73	18.9	137	35.5	25	6.5	156	40.4	23	6	
Rural	53	48.6	15	13.8	23	21.1	46	42.2	3	2.8	49	45.0	2	1.8	
Knowledge toward HIV															0.039
Low	124	54.6	26	11.5	46	20.3	81	35.7	10	4.4	93	41.0	8	3.5	
Moderate	93	47.9	16	8.2	33	17.0	73	37.6	9	4.6	86	44.3	10	5.2	
High	27	36.5	3	4.1	17	23.0	29	39.2	9	12.2	26	35.1	7	9.5	
Attitude Toward HIV															0.008
Negative	17	56.7	3	10.0	8	26.7	19	63.3	1	3.3	14	46.7	0	0.0	
Neutral	111	53.6	26	12.6	35	16.9	74	35.7	14	6.8	78	37.7	6	2.9	
Positive	116	45.0	16	6.2	53	20.5	90	34.9	13	5.0	113	43.8	19	7.4	
HIV Testing															0.751
Yes	52	45.2	9	7.8	20	17.4	39	23.9	9	7.8	46	40.0	7	6.1	
No	192	50.5	36	9.5	76	20.0	144	37.9	19	5.0	159	41.8	18	4.7	
Knowing infected HIV person															0.040
Yes	224	50.5	36	8.1	82	18.5	172	38.7	24	5.4	183	41.2	22	5.0	
No	20	39.2	9	17.6	14	27.5	11	21.6	4	7.8	22	43.1	3	5.9	
Ever Used Methamphetamine															0.334
Yes	60	56.1	7	6.5	20	18.7	39	36.4	5	4.7	37	34.6	3	2.8	
No	184	47.4	38	9.8	76	19.6	144	37.1	23	5.9	168	43.3	22	5.7	
Ever used alcohol or stimulant before sexual contact														0.048
Yes	94	48.5	11	5.7	40	20.6	77	39.7	8	4.1	78	40.2	8	4.1	
No	130	48.0	26	9.6	53	19.6	98	36.2	18	6.6	112	41.3	16	5.9	
Don't remember	20	66.7	8	26.7	3	10.0	8	26.7	2	6.7	15	50	1	3.3	
Ever used ecstasy															0.139
Yes	23	41.1	6	10.7	8	14.3	17	30.4	3	5.4	26	46.4	5	8.9	
No	212	50.4	33	7.8	85	20.2	160	38.0	23	5.5	171	40.6	20	4.8	
Don’t remember	9	50.0	6	33.3	3	16.7	6	33.3	2	11.1	8	44.4	0	0	
Age at first extramarital sexual contact															0.440
≤20	160	51.6	25	8.1	66	21.3	113	36.5	16	5.2	135	43.5	15	4.8	
>20	84	45.4	20	10.8	30	16.2	70	37.8	12	6.5	70	37.8	10	5.4	
Ever used injected Stimulant drugs															0.206
Yes	22	59.5	3	8.1	9	24.3	9	24.3	4	10.8	13	35.1	4	10.8	
No	222	48.5	42	9.2	87	19.0	174	38.0	24	5.2	192	41.9	21	4.6	

Reason 1: It was not accessible Reason 2: It was too expensive Reason 3: My partner objected Reason 4: I do not like it Reason 5: I used something else Reason 6: I did not think it was necessary Reason 7: Other

A high percentage of females thought condom use was not necessary (49.2%), but the most important reason for not using condoms in males was that condoms were not accessible (55.9%). Hence, the reasons for inconsistent condom use were different between females and males (*P*-value<0.0001).

People with low and moderate knowledge of HIV reported that ‘inaccessibility of condoms’ was the most significant reason for not using condoms (54.6% and 47.9%), respectively. On the other hand, people with high knowledge of HIV reported that they ‘did not like condom use’ as the most significant reason for not using condoms (39.2%). These differences between the three levels of knowledge of HIV was significant (*P*=0.039).

Three levels of attitude (negative, neutral, positive) towards HIV reported mostly the same reason for not using condoms—that it was not accessible (56.7%, 53.6%, and 45%)—and these differences were significant (*P*=0.008).

People who knew an infected HIV person reported the inaccessibility of condoms as the most important reason for inconsistent condom use (50.5%), but among people who did not know an infected HIV person, the most important reason was they did not think condoms were necessary (43.1%). Hence, these two groups had different reasons for not using condoms (*P*=0.040).

People who had used, not used, or did not remember using alcohol or stimulants before sexual contact, confirmed the inaccessibility of condoms as the most important reason for inconsistent condom use (48.5%, 48% and 66.7%), respectively. The differences observed between the three groups were significant (*P*=0.048). The relationship between other variables and reasons for inconsistent condom use were not significant at a level of 0.05.

Table 4 shows the adjusted odds ratio for choosing the reason ‘inaccessibility of condoms’ as a reason for inconsistent condom use in females vs. males was 0.33 (95% CI: 0.21, 0.51), those who ever used ecstasy compared to persons who had not used ecstasy was 0.55 (95% CI: 0.3, 0.99), and, in persons with high vs. low knowledge of HIV, it was 0.48 (95% CI: 0.27, 0.84). The adjusted odds ratio for selecting the reason ‘condom was too expensive’ as a reason for inconsistent condom use in persons who know vs. those who do not know an infected HIV person was 2.5 (95% CI: 1.03, 6.07), urban compared to rural was 0.48 (95% CI: 0.23, 0.99), age groups were 25–29 vs. 19–24 yr was 0.34 (95% CI: 0.16, 0.7), the age at first extramarital sexual contact >20 vs. ≤ 20 was 2.17 (95%CI:1.07, 4.3) and those who ever used ecstasy compared to persons who had not used ecstasy was 4.8 (95% CI: 1.45, 16.17). The variables gender, age and marital status had significant effects on choosing the reason ‘my partner objected’ as a reason for inconsistent condom use. Knowing an infected HIV person, gender, and attitude towards HIV exerted significant effects on choosing the reason ‘I do not like condoms’ as an excuse for inconsistent condom use. The variables knowledge of HIV and gender had significant effects on the selection of the excuses ‘I used something else’ and ‘I did not think a condom was necessary’ as reasons for inconsistent condom use respectively. None of the variables had significant effects on odds of choosing the reason ‘other’ for inconsistent condom use.

**Table 4:** Multivariate multilevel logistic regression for association between reasons for inconsistent condom use and independent variables

**Variables**	**Adjusted OR (95% CI)** ^a^	***P*** ** value**
Response: Condom was not accessible	
Gender		
Male	1.00	
Female	0.33(0.21, 0.51)	0.001
Ever used ecstasy		
No	1.00	
Yes	0.55 (0.30, 0.99)	0.040
Don’t remember	1.13 (0.41, 3.10)	0.800
Knowledge toward HIV		
Low	1.00	
Moderate	0.73 (0.49, 1.10)	0.140
High	0.48 (0.27, 0.84)	0.010
Response: Condom was too expensive	
Knowing infected HIV person		
No	1.00	
Yes	2.50 (1.03, 6.07)	0.040
Residence		
Rural	1.00	
Urban	0.48 (0.23, 0.99)	0.040
Age (yr)		
19-24	1.00	
25-29	0.34 (0.16, 0.70)	0.004
Age at first extramarital sexual contact	
≤ 20	1.00	
>20	2.17 (1.07, 4.30)	0.030
Ever used ecstasy		
No	1.00	
Yes	1.42 (0.54, 3.8)	0.470
Don’t remember	4.8 (1.45, 16.17)	0.010
Response: My partner objected		
Gender		
Male	1.00	
Female	2.41 (1.44, 4.03)	0.001
Age (yr)		
19-24	1.00	
25-29	0.35 (0.20, 0.59)	0.001
Marital status		
Single	1.00	
Married	0.57 (0.33, 0.98)	0.040
widowed/Divorced	0.75 (0.25, 2.23)	0.610
Response: I do not like condom		
Knowing infected HIV person		
No	1.00	
Yes	0.42 (0.20, 0.85)	0.010
Gender		
Male	1.00	
Female	0.50 (0.32, 0.79)	0.003
Attitude toward HIV		
Positive	1.00	
Negative	3.30 (1.15, 7.46)	0.004
Neutral	0.99 (0.67, 1.47)	0.990
Response: I used something else		
Knowledge toward HIV		
Low	1.00	
Moderate	1.05 (0.42, 2.65)	0.900
High	3.00 (1.17, 7.70)	0.020
Response: I did not think condom was necessary	
Gender		
Male	1.00	
Female	1.56 (1.02, 2.40)	0.030

^a^ The effect of each variable was adjusted by other significant variables in the model.

## Discussion

The use of condoms is a safe approach for preventing STI/HIV, but the prevalence of condom use in general, or in populations with high-risk behaviour, is low. Identifying the reasons for inconsistent condom use is important because through intervention, we can improve the consistency of condom use.

According to our findings, in those who had extramarital sex, 77.96% did not use condoms regularly. In Iran, 52.58% of homeless people with a history of illicit sex did not use condoms with regularity ^28^. This difference is because of the selected samples.

Our study showed the reason 'was not accessible condom' and ' condom use was not necessary ' were chosen mostly. Health policymakers should promote the knowledge of the general population, especially adult youths, about the advantages of condom use and expand the number of centres for distributing condoms to ensure accessibility. The item chosen the most for inconsistent condom use was related to partner relationships as trust in partner ^29^. The most important reason for consistent condom use was ‘avoidance pregnancy’^30^. The questionnaire did not include these items; hence, we could not compare these results with our findings.

In our study, the reasons for inconsistent condom use were different in two groups of ages 19–24 and 25–29 yr. More than the age group 25–29 yr, adults aged 19–24 yr reported that condoms were too expensive. This is possibly caused by the fact that most of them are students and are unemployed. Adults aged 25–29 yr noted more the objections of the partner as a reason for inconsistent condom use. The selection of this reason arises from low knowledge of STI or HIV; hence, it is necessary to improve this by educating youths in schools and universities. The other reasons chosen were nearly identical in the two groups.

Men reported the reasons for inconsistent condom use more than females in the choices: ‘inaccessibility of condoms’ and ‘do not like the use of condoms’. This difference was because of when a man did not like condom, the female partner usually accepted it because she wanted to pay attention to the man, according to cultural norms.

The reasons for inconsistent condom use were different at levels of knowledge towards HIV: For example, adults with low levels of knowledge mostly selected the item ‘inaccessibility of condom’ while adults with high levels of knowledge selected the reason ‘do not like condom use’.

Health policymakers should make adults aware about STI/HIV, make accessibility to condoms easier, and explain to them the places where condoms are distributed. They should use methods for attracting condoms in others to encourage them for using condom and like to use it.

The reason ‘don’t like condoms’ was frequently selected by adults with a negative attitude towards HIV, but in people with a positive attitude, ‘inaccessibility to condoms’ was frequently selected. This difference showed that adults with a negative attitude prefer emotions than dangerous of STI/HIV. Therefore, by making clips and posters and advertisements on television or billboards in the city, we can improve the attitude of adults towards the ways of transmission of HIV.

Our results showed that the selections of the reasons for inconsistent condom use were different among those who know/do not know a person among family members, relatives or friends who were infected by HIV. This result is consistent with that of a study in Iran, which showed there was a statistically significant relationship between the awareness of current HIV status with consistent condom use ^31^.

Adults who reported that they used stimulant(s) or alcohol before sexual contact frequently selected ‘inaccessibility of condoms’. The use of alcohol or stimulant(s) disturbs the operations of the brain and causes risky behaviour, such as unsafe sexual contact.

In Iran, higher educational levels, perceived HIV risk and perceived family intimacy during childhood were associated with lower odds of having unprotected sex with IDU(s) in the month preceding the study ^32^. Our study was conducted in the general population over the ages of 19 to 29 yr; hence, the levels of education did not affect the reasons for inconsistent condom use.

Based on a study conducted among Iranian male drug injectors, “the odds of inconsistent condom use were higher among those with a history of syringe sharing, but lower among those with higher education levels, those who mostly inject at home, and those with a history of treatment” ^13^. Our results also showed adults with risky behaviour such as those using alcohol or stimulant(s) before sexual contact had different reasons for inconsistent condom use compared to adults without risky behaviour.

The results of condom use in regular female sex partners by male drug users in South Asia showed that the use of drugs before the last sexual contact, familiarity with HIV/AIDS, the fact that they had ever used drugs, and were related to someone who has information on HIV were common in those likely to have used condoms during the last sexual contact, about twice or more. In addition, condom use was less in women who had a single partner ^33^.

Modelling the reasons for inconsistent condom use helps us identify those groups of adults, which have greater odds for choosing each reason. Health makers should decide to provide accessibility to condoms in males and adults with low knowledge of HIV. Distributing condoms freely among rural adults aged 19–24 yr, adults who used ecstasy, and those knowing an infected HIV person can help them preclude inconsistent condom use because condoms are expensive

## Limitations

The sampling method was street-based. The nature of this sampling may introduce bias into our results and limit its generalizability to some extent. In Iran the answers to sensitive questions were reliable when the sampling method was street-based instead of household-level and telephone-based surveys,^35^.

## Conclusions

Most of the reasons selected for insistent condom use were ‘inaccessibility to condoms’ and ‘not knowing the essentials of it’. Hence, it is important to improve the knowledge of adults towards STI/HIV and expand the locations of condom distribution. We should try to promote the culture of condom use as a routine. Unfortunately, currently in Iran, people are ashamed to demand condoms because other people look at them in an embarrassing manner.

## Acknowledgment

The authors would like to thank the study participants for their contribution to the research.

## Conflict of interest statement

The authors declare no conflicts of interest

## Funding

This survey was financially supported by the Ministry of Health of Iran in 2013.

## Highlights

- The prevalence of inconsistent condom use was considerable.
- The reason frequently chosen for inconsistent condom use was inaccessibility of it.
- The effect of gender was significant on most of reasons for inconsistent condom use.
